# Complicated Carriage with Methicillin-Resistant Staphylococcus aureus: Evaluation of the Effectiveness of Decolonization Regimens Advised in the Dutch National Guideline

**DOI:** 10.1128/AAC.00257-21

**Published:** 2021-08-17

**Authors:** A. C. Westgeest, E. F. Schippers, N. M. Delfos, L. J. Ellerbroek, T. Koster, V. Hira, L. G. Visser, M. G. J. de Boer, M. M. C. Lambregts

**Affiliations:** a Department of Infectious Diseases, Leiden University Medical Center, Leiden, The Netherlands; b Department of Internal Medicine, Haga Teaching Hospital, The Hague, The Netherlands; c Department of Internal Medicine, Alrijne Hospital, Leiderdorp, The Netherlands; d Department of Internal Medicine, Reinier de Graaf Hospital, Delft, The Netherlands; e Department of Internal Medicine, Groene Hart Ziekenhuis, Gouda, The Netherlands; f Department of Medical Microbiology and Infection Prevention, Groene Hart Ziekenhuis, Gouda, The Netherlands

**Keywords:** MRSA, MRSA carriage, decolonization, eradication treatment, methicillin-resistant *Staphylococcus aureus*

## Abstract

Methicillin-resistant Staphylococcus aureus (MRSA) colonization leads to increased infection rates and mortality. Decolonization treatment has been proven to prevent infection and reduce transmission. As the optimal antimicrobial strategy is yet to be established, different regimens are currently prescribed to patients. This study aimed to evaluate the efficacy of the decolonization treatments recommended by the Dutch guideline. A retrospective multicenter cohort study was conducted in five Dutch hospitals. All patients who visited the outpatient clinic because of complicated MRSA carriage between 2014 and 2018 were included. We obtained data on patient characteristics, clinical and microbiological variables relevant for MRSA decolonization, environmental factors, decolonization regimen, and treatment outcome. The primary outcome was defined as three negative MRSA cultures after treatment completion. Outcomes were stratified for the first-line treatment strategies. A total of 131/224 patients were treated with systemic antibiotic agents. Treatment was successful in 111/131 (85%) patients. The success rate was highest in patients treated with doxycycline-rifampin (32/37; 86%), but the difference from any of the other regimens did not reach statistical significance. There was no difference in the success rate of a 7-day treatment compared to that with 10 to 14 days of treatment (odds ratio [OR], 0.99; 95% confidence interval [CI], 0.39 to 2.53; *P* = 1.00). Side effects were reported in 27/131 (21%) patients and consisted mainly of mild gastrointestinal complaints. In a multivariable analysis, an immunocompromised status was an independent risk factor for failure at the first treatment attempt (OR, 4.65; 95% CI, 1.25 to 17.25; *P* = 0.02). The antimicrobial combinations recommended to treat complicated MRSA carriage yielded high success rates. Prolonged treatment did not affect treatment outcome. A randomized trial is needed to resolve whether the most successful regimen in this study (doxycycline plus rifampin) is superior to other combinations.

## INTRODUCTION

Methicillin-resistant Staphylococcus aureus (MRSA) is a challenging global health problem. Colonization with MRSA leads to increased infection risks, which range from mild skin infections to severe clinical syndromes, i.e., pneumonia and bloodstream infection (1–3). Mortality is high in MRSA infections compared to that in infections caused by their more susceptible counterparts (4). This may in part be attributed to decreased antibiotic effectiveness and increased toxicity of the antibiotic therapy.

Decolonization of MRSA in carriers has proven to be an effective preventive strategy in reducing infection and hospitalization rates (5, 6). In Europe, the prevalence of MRSA in Staphylococcus aureus blood isolates was 16.4% in 2018, with large intercountry variation (7). In the Netherlands, the MRSA prevalence in blood culture isolates is 1.4%, which is, along with that of the Scandinavian countries, one of the lowest in the world (7, 8). The low prevalence in the Netherlands is to a large part ascribed to the “search and destroy policy” targeting MRSA carriers (9–11). The aim of this policy is to minimize colonization and transmission in both health care workers (HCWs) and patients. Active screening (e.g., after hospitalization abroad), isolation of MRSA carriers, and preemptive isolation of risk groups are part of this policy (11). The policy also urges decolonization treatment in all MRSA carriers.

The Dutch guideline for the treatment of MRSA carriage differentiates between complicated and uncomplicated carriership (12). Uncomplicated carriership, i.e., exclusively located in the nose and without active infection, is advised to be treated with topical therapy (mupirocin topically applied to the nares) and hygienic measures. In cases of complicated MRSA carriage, additional systemic antimicrobial therapy with a combination of two antibiotic agents is recommended (see Table 1). Due to the limited availability of data (13–17), it has remained undecided which combination of antistaphylococcal agents is most effective. The individual treatment regimen, i.e., the choice of antibiotic agents and the treatment duration in clinical practice, is therefore variable (18). The aim of this study was to describe the effectiveness of different MRSA decolonization treatments for complicated MRSA carriage.

**TABLE 1:** Oral antibiotic combination therapy for decolonization of MRSA colonization according to the Dutch national guideline

Therapy	Antibiotic agent^*a*^	
	1	2
Recommended	Doxycycline 200 mg qd or Trimethoprim 200 mg bid	Rifampin 600 mg bid
Alternative	Clindamycin 600 mg tid or clarithromycin 500 mg bid or ciprofloxacin 750 mg bid or fusidic acid 500 mg tid	Fusidic acid 500 mg tid

a qd, once a day; bid, twice a day; tid, three times a day.

## RESULTS

**FIG 1:**
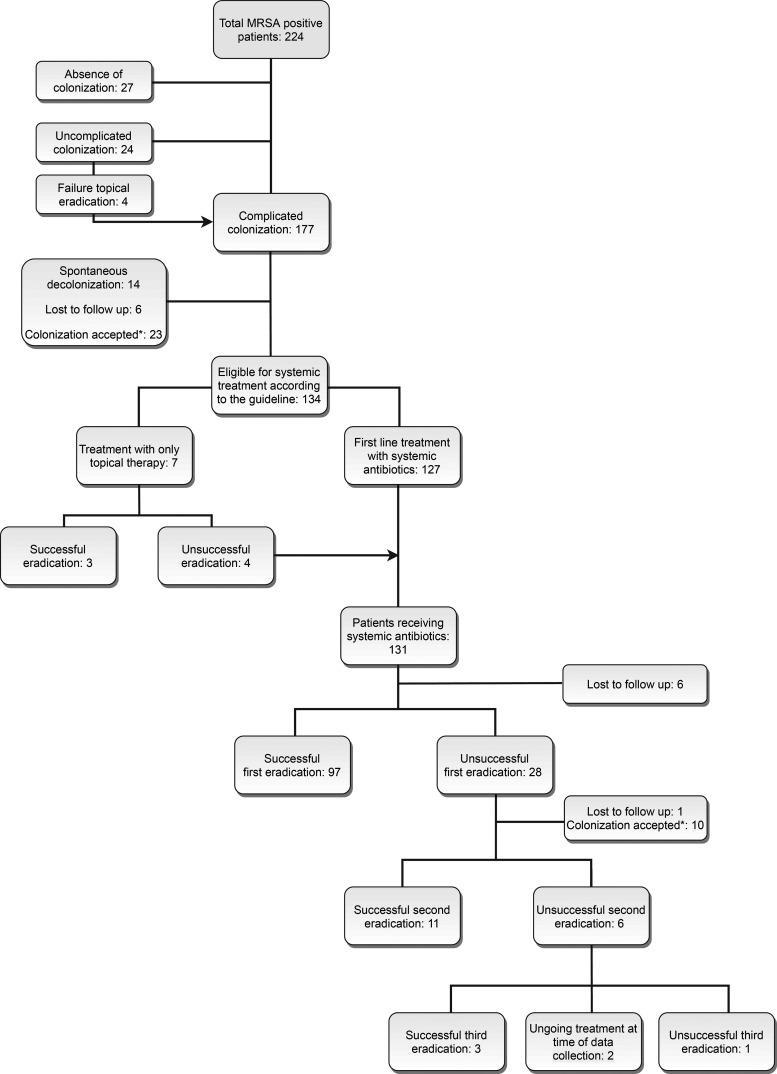
Flowchart of treatment schedule. Uncomplicated MRSA carriership was defined as the presence of all of the following features: (i) MRSA exclusively located in the nose, (ii) no active infection with MRSA, (iii) *in vitro* sensitivity for mupirocin, (iv) the absence of active skin lesions, (v) the absence of foreign material that connects an internal body site with the outside (e.g., urine catheter, external fixation material), and (vi) no previous failure of decolonization treatment. All other cases were considered complicated. Successful decolonization was defined by three successive negative MRSA swabs from nose, throat, and perineum at least 48 h after treatment, with a minimum interval of 1 week. An asterisk (*) indicates that colonization was accepted under certain circumstances, e.g., active noncurable skin lesions, short life expectancy, wishes of the patient, or a high risk of recurrence due to frequent livestock contact or regular visits to health care facilities abroad. An arrowhead indicates patients added to another group.

During the study period, 224 patients were referred to the outpatient departments because of MRSA colonization. Because of the absence of colonization or uncomplicated carriership at the first evaluation, 27 and 20 patients, respectively, were excluded. Of the remaining 177 patients, only 131 received systemic antibiotics (Fig. 1). Reasons for not starting decolonization with systemic antibiotics were spontaneous clearance of colonization (14/177; 8%), lost to follow-up (6/177; 3%), and/or acceptance of colonization (23/177; 13%). Reasons for accepting colonization were either related to a high risk of failure, i.e., therapy-resistant skin lesions in eczema, or to a high risk of recurrence, i.e., frequent livestock contact or regular visits to health care facilities abroad. Three patients (3/177; 2%) were successfully treated with topical therapy only.

The patient characteristics of all 177 patients with complicated colonization and those of the 131 patients with complicated colonization that were treated with systemic antibiotic therapy are summarized in Table 2.

**TABLE 2:** Patient characteristics^*a*^

Characteristic	All patients (*N* = 177) with complicated MRSA colonization	Patients (*N* = 131)^*c*^ receiving treatment with systemic antibiotics
Sex, male (no. [%])	82 (46)	64 (49)
Age (yrs) (median [IQR])	41 (12–70)	43 (13–73)
Positive household member (no. [%])	76 (43)	61 (47)
Risk factors for colonization (no. [%])		
Immunocompromised status	17 (10)	12 (9)
Chronic antibiotic use	7 (4)	7 (5)
Health care worker	27 (15)	22 (17)
Professional livestock contact	4 (2)	3 (2)
Reason for MRSA screening prior to referral (no. [%])		
Positive household member	44 (25)	29 (22)
Contact with positive person in health care facility	32 (18)	26 (20)
Infection with MRSA	59 (33)	42 (32)
Screening after contact with livestock	2 (1)	0
Screening after foreign hospital	25 (14)	22 (17)
Other	8 (5)	7 (5)
Unknown	7 (4)	4 (3)
Site of colonization (no. [%])		
Nose	118 (67)	88 (67)
Throat	114 (64)	87 (66)
Perineum	98 (55)	70 (53)
Other (e.g., skin lesions, infection sites)	58 (33)	40 (31)
Reason for complicated colonization (no. [%])		
Extranasal colonization	166 (94)	122 (93)
Foreign material internal-external	6 (3)	2 (2)
Mupirocin resistance	4 (2)	4 (3)
Skin lesions	33 (19)	24 (18)
Previous unsuccessful decolonization	20 (11)	14 (11)
MRSA infection during colonization^*b*^ (no. [%])	65 (37)	45 (34)
Microbiology results (no. [%])		
PVL		
Present	36 (20)	27 (21)
Absent	78 (44)	61 (47)
NA	63 (36)	43 (32)
Rifampin		
Susceptible	158 (89)	119 (91)
Resistant	4 (2)	4 (3)
NA	15 (9)	8 (6)
Trimethoprim-sulfamethoxazole susceptible		
Susceptible	136 (77)	103 (79)
Resistant	27 (15)	20 (15)
NA	14 (8)	8 (6)
Clindamycin		
Susceptible	111 (63)	79 (60)
Resistant	43 (24)	36 (28)
NA	23 (13)	16 (12)
Doxycycline		
Susceptible	72 (41)	60 (46)
Resistant	38 (22)	28 (21)
NA	67 (37)	43 (33)

a Values are counts (%) for categorical variables and median (interquartile range [IQR]) for continuous variables. PVL, Panton-Valentine leucocidin; NA, not applicable.

b Defined as culture-confirmed infection(s) with MRSA during colonization.

c Out of the total of 177 patients with complicated MRSA, 131 received treatment with systemic antibiotics.

Of the 131 patients with complicated colonization and treatment with systemic antibiotics, 19 (15%) lived alone, 103 (79%) lived with one or more household members, and for 9 patients (7%) data on household members were missing. In the cases of 91/103 (88%) patients, all household members were screened for carriership. In 5/103 (5%) cases only some of the household members were screened, and in 7/103 (7%) none of the household members were screened. In total, 229 household members were screened, of which 91 (40%) tested positive for MRSA.

### Decolonization treatment.

In 131 patients, systemic antibiotic treatment was prescribed (Fig. 1), and in 125/131 (95%) the choice of antibiotic regimen was in line with the national guideline (Table 1). Six patients received antimicrobial combinations that were not in line with the guideline, and 4 others were initially treated with hygienic measures and topical therapy only.

The success rate of the first decolonization attempt was 97/131 (74%). Not all patients that failed on a first treatment were treated again. Of the 34 patients in whom the first decolonization attempt failed, 17/34 (50%) underwent a second treatment (Table 3). The success rate after this second treatment was 11/17 (65%). Of the remaining six patients, four were treated for a third time, which was successful in 3/4 (75%) of patients. The cumulative success rate was 111/131 (85%). Mean follow-up time was 13 months. In 78/111 (70%) of the initially successfully treated patients, follow-up cultures at a time (T) of ≥12 months were available. In 4/78 (5%) of patients, these cultures were positive for MRSA. Side effects were reported in 27/131 (21%) of patients and consisted of gastrointestinal complaints (21/131; 16%) and malaise (4/131; 3%). An allergic reaction occurred in 1 of the 131 patients.

**TABLE 3:** Follow-up cultures after decolonization treatment^*a*^

Decolonization attempt	Follow-up culture after treatment	No. of patients (*N* = 131) at:		
		Wk 1	Wk 2	Wk 3
First	Available	130	111	103
	Positive	14	8	6
Second	Available	17	15	13
	Positive	2	2	2
Third	Available	4	4	3
	Positive	0	1	0

a After one positive culture, no further follow-up cultures were performed.

### Antibiotic regimens.

For the treatment of complicated colonization in this cohort, 12 different combinations of antibiotic agents were prescribed with a duration ranging from 5 to 14 days. The most frequently prescribed combinations of antibiotic agents were doxycycline-rifampin, trimethoprim (with or without sulfamethoxazole)-rifampin, and clindamycin-rifampin. The success rates of the different antibiotic combinations at the consecutive decolonization attempts are summarized in Table 4. In the first treatment attempt, the combination of doxycycline-rifampin showed the highest success rate (32/37; 86%) compared to those of trimethoprim(-sulfamethoxazole)-rifampin (41/60; 68%), clindamycin-rifampin (15/19; 79%) and “other regimens” (9/15; 60%). The difference in success rate at first attempt of doxycycline-rifampin versus those of all other regimens did not reach statistical significance (86% versus 69%; odds ratio [OR], 2.20; 95% confidence interval [CI], 0.77 to 6.31; *P* = 0.16). There was no difference between outcomes with addition of trimethoprim alone (success rate, 19/24 [79%]; 95% CI, 58 to 93) or in combination with sulfamethoxazole (success rate, 22/31 [71%]; 95% CI, 52 to 86).

**TABLE 4:** Decolonization success rates of antibiotic regimens

Antibiotic agents^*a*^	No. treated first attempt^*c*^	No. successful after first attempt (%; 95% confidence interval)	No. treated second attempt	No. (%) successful after second attempt	No. treated third attempt	No. (%) successful after third attempt
Doxycycline plus rifampin	37	32 (86; 71–96)	1	1	0	
Trimethoprim^*b*^ plus rifampin	60	41 (68; 55–80)	8	5	3	2
Clindamycin plus rifampin	19	15 (79; 54–94)	2	1	1	1
Other	15	9 (60; 32–84)	6	4	0	
Total	131	97 (74)	17	11 (65)	4	3 (75)

a The most frequently used combinations of antibiotic agents are shown separately, and the 8 other antibiotic regimens are bundled in “other.”

b Trimethoprim was with or without sulfamethoxazole.

c “First attempt” is defined as the first attempt with systemic antibiotic agents added to the treatment, i.e., the first treatment episode in complicated colonization or the second treatment episode after failure of first treatment with topical treatment in uncomplicated colonization.

Prolonged antibiotic treatment (10 to 14 days) was not associated with a better treatment outcome (49/64; 77%) compared to a 7-day treatment (40/51; 78%) (OR, 0.99; 95% CI, 0.39 to 2.53; *P* = 1.00). There was a trend toward a higher success rate in the patients in whom the guideline for treatment choice was followed (88/115; 77%) compared to that in the patients in whom the guideline was not followed (6/12 [50%]; 95% CI, 0.97 to 10.94; *P* = 0.08).

### Predictive variables.

In the univariate risk analysis, being part of a known household cluster (OR, 2.38; 95% CI, 1.01 to 5.61; *P*= 0.05) and an immunocompromised status (OR, 6.27; 95% CI, 1.81 to 21.68; *P* < 0.01) were associated with failure at first decolonization attempt (Table 5).

**TABLE 5:** Univariate and multivariable analysis of predictive variables for failure of first decolonization attempt

Variable	Univariate analysis		Multivariable analysis		
	OR (95% CI)	*P* value	B^*a*^	OR (95% CI)	*P* value
Patient characteristics					
Age, >60 yrs	0.68 (0.23–1.98)	0.61			
Gender, male	1.54 (0.66–3.58)	0.39			
Part of a known household cluster	2.38 (1.01–5.61)	0.05	0.60	1.83 (0.74–4.51)	0.19
Healthcare worker	0.54 (0.15–1.99)	0.56			
Comorbidities					
Immunocompromised status	6.27 (1.81–21.68)	<0.01	1.58	4.83 (1.34–17.45)	0.02
Current skin disease	0.66 (0.21–2.11)	0.59			
Chronic antibiotic use	1.83 (0.32–10.53)	0.61			
MRSA infection^*b*^	1.29 (0.54–3.08)	0.65			
Site of colonization other than nose^*c*^					
Throat culture positive	0.84 (0.34–2.11)	0.81	0.07	1.07 (0.39–2.96)	0.89
Perineum culture positive	1.51 (0.62–3.71)	0.39	0.40	1.49 (0.57–3.90)	0.42
Other site culture positive	1.20 (0.49–2.97)	0.81			
Positive for PVL^*d*^ genes	1.56 (0.49–4.93)	0.54			

a B, regression coefficient.

b MRSA infection was defined as culture-confirmed infection(s) with MRSA during colonization.

c Sites of colonization reflects positive cultures at screening. Multiple sites could be positive within one patient.

d PVL, Panton-Valentine leucocidin.

Panton-Valentin leucocidin (PVL) was tested in 88 patients and was positive in 27/88 (31%). There was no correlation between PVL positivity and success of eradication in these patients (OR, 0.57; 95% CI, 0.15 to 1.82; *P* = 0.36).

In the multivariable analysis, an immunocompromised status remained an independent risk factor for failure at the first treatment attempt (OR, 4.65; 95% CI, 1.25 to 17.25; *P* = 0.02) (Table 5).

## DISCUSSION

The main finding of our study is the success rate of decolonization of 74% after the first treatment attempt, which is relatively high compared to those reported in previous literature. In the Dutch study by Ammerlaan et al. in 2011, this rate was 56% (18). A possible explanation for this difference may be that the guideline adherence for treatment choice was much lower in the study by Ammerlaan (62%) than that in our study (90%). A second explanation may be that in our study—in the majority of cases—household members were screened and treated simultaneously, preventing failure because of recolonization by untreated colonized household contacts. At the time of the study by Ammerlaan et al., according to the Dutch guideline, household members were only screened if the first decolonization attempt had failed. Routine screening of household members before starting treatment was not included in the guideline until 2012.

The success rate of topical treatment in combination with systemic antibiotics in our study is decidedly high compared to that of topical treatment without systemic antibiotics reported in the literature, supporting the current guideline. Earlier studies have shown a success rate of approximately 40% after the first decolonization attempt in patients that were treated with topical treatment alone (19, 20).

There were no apparent differences in success rates between different antibiotic regimens. The combination of doxycycline-rifampin had the highest success rate, but this did not reach statistical significance. This combination is one of the first-choice regimens in the Dutch guideline. There was no difference in effectivity between a treatment duration of 7 days and a duration of 10 to 14 days. This supports the guideline recommendation of a minimum antibiotic treatment of 7 days (12).

Being part of a known household cluster and immunocompromised status were associated with failure at the first treatment attempt. In the multivariable analysis, only immunocompromised status remained an independent risk factor for failure at the first treatment attempt, although there were few patients (12) in this group. This differs from an earlier study by Ammerlaan et al., in which chronic pulmonary disease, activities of daily living (ADL) dependency, throat carriage, perineal carriage, and the presence of a device were associated with treatment failure (20). This difference may be explained by the difference in study population, as Ammerlaan et al. did not exclude uncomplicated carriers from their analyses.

The fact that 27/224 (12%) of the referred patients were no longer colonized with MRSA at the time of visiting the outpatient clinic is a relevant observation. It illustrates the possibility of spontaneous clearance and the importance of repeated screening before starting treatment.

In the current search and destroy strategy, MRSA carriers are exposed to systemic antibiotic therapy, for the benefit of society, even if they are asymptomatic. The side effects of treatment should be weighed against the benefits of a search and destroy policy. Reported side effects in this study were mild and the effectivity of decolonization was high, supporting the current MRSA decolonization strategy in a low-prevalence country like the Netherlands.

There are several limitations of our study. Due to its observational design, confounding limits the determination of the most effective antibiotic strategy. However, so far, there has only been one small randomized trial published comparing the efficacy of ciprofloxacin-rifampin and trimethoprim-sulfamethoxazole combinations in MRSA decolonization. This study showed no significant difference in success rates, but it did not include a doxycycline-based regimen and was underpowered (14). The majority of previously published studies are limited to the comparison of different antibiotic combinations versus topical treatment alone or no treatment at all (15, 17).

A second limitation of our study is that group sizes are small due to the low prevalence of MRSA colonization and the variety of different antibiotic regimens that were prescribed, reflecting the current guideline.

A third limitation is that a proportion of patients were lost to follow-up 1 year after treatment. However, only 5% of the initially successfully treated patients that were cultured after 1 year were recolonized with MRSA. In the study by Lekkerkerk et al. (22), the median number of days to detect a MRSA recurrence was 24, and 12% of recurrences was detected between 62 and 200 days. Therefore, the majority of recurrences are expected to have been detected in our study, but late recurrences may have been missed. However, these late recurrences could also be ascribed to recolonization from an unidentified source rather than to failure of the initial decolonization treatment.

In conclusion, treatment for complicated MRSA colonization according to the guideline has a high success rate. These findings endorse the current strategy of “search and destroy.” For future research, a randomized trial would be necessary to further distinguish whether doxycycline-rifampin has a higher efficacy rate than those of alternative treatment combinations, as suggested in this study.

## MATERIALS AND METHODS

A multicenter retrospective cohort study was conducted in five Dutch hospitals (one university hospital and four large regional teaching hospitals).

### Study population.

All consecutive patients referred to the outpatient clinic with complicated MRSA colonization from January 2014 until December 2018 were eligible for inclusion. Exclusion criteria were the absence of MRSA colonization upon screening at the outpatient clinic, uncomplicated carriership, and/or a patient’s objection to the use of their medical file for research purposes.

### Outpatient clinic.

History taking and physical examination were performed during the first visit to the outpatient clinic. Physical examination included skin examination, as skin lesions such as eczema may impede effective decolonization. Furthermore, physical examination involved examination of the oral cavity. Culture swabs were routinely obtained from nose, throat, and perineum. If skin lesions, e.g., wounds were present, additional cultures were obtained from these sites. Household contacts were screened as well, and colonized household contacts were treated simultaneously and were included in the study. The standard treatment consisted of nasal mupirocin thrice daily, topical disinfectants daily (chlorhexidine soap and betadine shampoo), and hygienic measures. Hygienic measures included daily changing of underwear, clothes, and towels, as well as changing of bed linen on days 1, 2, and 5. The first-choice recommended systemic antibiotic agent combinations were doxycycline-rifampin and trimethoprim-rifampin, according to *in vitro* susceptibility (12). Alternative combinations were either (i) rifampin or fusidic acid in combination with clindamycin, clarithromycin, or ciprofloxacin or (ii) rifampin and fusidic acid (Table 1). The standard duration of antibiotic treatment was a minimum of 7 days.

### Microbiological methods.

Culturing and susceptibility determination was performed according to the Dutch Society of Medical Microbiology guideline for laboratory detection of highly resistant microorganisms. MIC breakpoints and zone diameter breakpoints for resistance and intermediate sensitivity were based on EUCAST criteria (23).

### Data collection.

The electronic patient files were reviewed to record patient characteristics, clinical data relevant for MRSA decolonization (e.g., immune status and skin diseases), environmental factors (e.g., health care profession, household members), and microbiological data (culture results and antimicrobial susceptibility patterns). In each hospital, the prescribed antibiotic therapy and treatment duration for all treatment episodes were extracted from the hospital’s electronic prescribing system. Microbiological data were retrieved from the Department of Medical Microbiology of each hospital.

### Definitions.

Uncomplicated MRSA carriership was defined as having all of the following features: (i) the presence of MRSA exclusively located in the nose, (ii) no active infection with MRSA, (iii) *in vitro* sensitivity for mupirocin, (iv) the absence of active skin lesions, (v) the absence of foreign material that connects an internal body site with the outside (e.g., urine catheter, external fixation material), and (vi) no previously failure of decolonization treatment. All other situations were considered complicated colonization (12).

An “isolated patient” was defined as a solitary carrier without any known family or household members with MRSA colonization. In the case of any known positive family or household member, these patients together were considered a cluster. A household member was defined as a person sharing the same house by day and night and sharing a bedroom and/or bathroom and/or living room and/or kitchen (12).

Immunocompromised status was defined as either a hematologic malignancy, stem cell transplantation, organ transplantation, immunosuppressive medication (e.g., chemotherapy, steroids), or HIV infection.

The primary outcome of the study was the success rate of decolonization treatment, defined by three successive negative MRSA cultures from swabs taken from nose, throat, and perineum. The first culture needed to be taken at least 48 h after treatment, with the follow-up cultures obtained at 1-week intervals. The long-term success rate was defined as an additional set of negative MRSA swabs 1 year after decolonization treatment (data available for four hospitals).

### Statistical analysis and outcome.

Data are presented as rates (percentages or proportions) for categorical variables and as medians plus interquartile range (IQR) for continuous variables. The overall success rate of decolonization treatment is presented as a rate, with 95% confidence interval, and is stratified for different treatment strategies.

In univariate analysis, odds ratios (with 95% confidence intervals) and Fisher’s exact tests were applied to identify clinical risk factors of treatment failure. In the multivariable regression analysis, variables with a *P* value of <0.05 in the univariate analysis were included, together with variables that were previously reported to be associated with treatment failure, namely, MRSA throat carriage and perineal carriage (19, 21).

### Ethical approval.

Ethical approval was granted by the institutional ethical review committee of the Leiden University Medical Center and the participating hospitals.

## References

[1] European Centre for Disease Prevention and Control. 2019. Surveillance of antimicrobial resistance in Europe, 2018. European Centre for Disease Prevention and Control, Stockholm, Sweden.

[2] de Greeff SC, Mouton JW, Schoffelen AF, Verduin CM. 2019. NethMap 2019: Consumption of antimicrobial agents and antimicrobial resistance among medically important bacteria in the Netherlands/MARAN 2019: monitoring of antimicrobial resistance and antibiotic usage in animals in the Netherlands in 2018. Rijksinstituut voor Volksgezondheid en Milieu RIVM Stichting Werkgroep Antibiotica Beleid SWAB, Bilthoven, the Netherlands.

[3] Davis KA, Stewart JJ, Crouch HK, Florez CE, Hospenthal DR. 2004. Methicillin-resistant *Staphylococcus aureus* (MRSA) nares colonization at hospital admission and its effect on subsequent MRSA infection. Clin Infect Dis 39:776–782. doi:10.1086/422997. 15472807

[4] von Eiff C, Becker K, Machka K, Stammer H, Peters G. 2001. Nasal carriage as a source of *Staphylococcus aureus* bacteremia. N Engl J Med 344:11–16. doi:10.1056/NEJM200101043440102. 11136954

[5] Wenzel RP, Perl TM. 1995. The significance of nasal carriage of *Staphylococcus aureus* and the incidence of postoperative wound infection. J Hosp Infect 31:13–24. doi:10.1016/0195-6701(95)90079-9. 7499817

[6] Cosgrove SE, Sakoulas G, Perencevich EN, Schwaber MJ, Karchmer AW, Carmeli Y. 2003. Comparison of mortality associated with methicillin-resistant and methicillin-susceptible *Staphylococcus aureus* bacteremia: a meta-analysis. Clin Infect Dis 36:53–59. doi:10.1086/345476. 12491202

[7] Huang SS, Singh R, McKinnell JA, Park S, Gombosev A, Eells SJ, Gillen DL, Kim D, Rashid S, Macias-Gil R, Bolaris MA, Tjoa T, Cao C, Hong SS, Lequieu J, Cui E, Chang J, He J, Evans K, Peterson E, Simpson G, Robinson P, Choi C, Bailey CC, Leo JD, Amin A, Goldmann D, Jernigan JA, Platt R, Septimus E, Weinstein RA, Hayden MK, Miller LG; Project CLEAR Trial. 2019. Decolonization to reduce postdischarge infection risk among MRSA carriers. N Engl J Med 380:638–650. doi:10.1056/NEJMoa1716771. 30763195 PMC6475519

[8] Kluytmans JAJW, Mouton JW, VandenBergh MFQ, Manders M-JAAJ, Maat APWM, Wagenvoort JHT, Michel MF, Verbrugh HA. 1996. Reduction of surgical-site infections in cardiothoracic surgery by elimination of nasal carriage of *Staphylococcus aureus*. Infect Control Hosp Epidemiol 17:780–785. doi:10.1017/S0195941700003465. 8985763

[9] Vos MC, Behrendt MD, Melles DC, Mollema FPN, de Groot W, Parlevliet G, Ott A, Horst-Kreft D, van Belkum A, Verbrugh HA. 2009. 5 Years of experience implementing a methicillin-resistant *Staphylococcus aureus* search and destroy policy at the largest university medical center in the Netherlands. Infect Control Hosp Epidemiol 30:977–984. doi:10.1086/605921. 19712031

[10] Wertheim HFL, Vos MC, Boelens HAM, Voss A, Vandenbroucke-Grauls CMJE, Meester MHM, Kluytmans JAJW, van Keulen PHJ, Verbrugh HA. 2004. Low prevalence of methicillin-resistant *Staphylococcus aureus* (MRSA) at hospital admission in the Netherlands: the value of search and destroy and restrictive antibiotic use. J Hosp Infect 56:321–325. doi:10.1016/j.jhin.2004.01.026. 15066745

[11] Dutch Working Party on Infection Prevention. 2017. Guideline MRSA, hospitals. WIP-richtlijn, The Netherlands.

[12] Dutch Working Party on Antibiotic Policy (Stichting Werkgroep Antibiotica Beleid [SWAB]). 2012. Guideline for the treatment of MRSA carriage. Secretariaat SWAB, Nijmegen, the Netherlands.

[13] Ammerlaan HS, Kluytmans JA, Wertheim HF, Nouwen JL, Bonten MJ. 2009. Eradication of methicillin-resistant *Staphylococcus aureus* carriage: a systematic review. Clin Infect Dis 48:922–930. doi:10.1086/597291. 19231978

[14] Peterson LR, Quick JN, Jensen B, Homann S, Johnson S, Tenquist J, Shanholtzer C, Petzel RA, Sinn L, Gerding DN. 1990. Emergence of ciprofloxacin resistance in nosocomial methicillin-resistant *Staphylococcus aureus* isolates: resistance during ciprofloxacin plus rifampin therapy for methicillin-resistant *S aureus* colonization. Arch Intern Med 150:2151–2155. doi:10.1001/archinte.1990.00390210111024. 2222100

[15] Simor AE, Phillips E, McGeer A, Konvalinka A, Loeb M, Devlin HR, Kiss A. 2007. Randomized controlled trial of chlorhexidine gluconate for washing, intranasal mupirocin, and rifampin and doxycycline versus no treatment for the eradication of methicillin-resistant *Staphylococcus aureus* colonization. Clin Infect Dis 44:178–185. doi:10.1086/510392. 17173213

[16] Walsh TJ, Standiford HC, Reboli AC, John JF, Mulligan ME, Ribner BS, Montgomerie JZ, Goetz MB, Mayhall CG, Rimland D. 1993. Randomized double-blinded trial of rifampin with either novobiocin or trimethoprim-sulfamethoxazole against methicillin-resistant *Staphylococcus aureus* colonization: prevention of antimicrobial resistance and effect of host factors on outcome. Antimicrob Agents Chemother 37:1334–1342. doi:10.1128/AAC.37.6.1334. 8328783 PMC187962

[17] Lindgren AK, Nilsson AC, Åkesson P, Gustafsson E, Melander E. 2018. Eradication of methicillin-resistant *Staphylococcus aureus* (MRSA) throat carriage: a randomised trial comparing topical treatment with rifampicin-based systemic therapy. Int J Antimicrob Agents 51:642–645. doi:10.1016/j.ijantimicag.2017.08.021. 28843819

[18] Ammerlaan HSM, Kluytmans JAJW, Berkhout H, Buiting A, de Brauwer EIGB, van den Broek PJ, van Gelderen P, Leenders SACAP, Ott A, Richter C, Spanjaard L, Spijkerman IJB, van Tiel FH, Voorn GP, Wulf MWH, van Zeijl J, Troelstra A, Bonten MJM; MRSA Eradication Study Group. 2011. Eradication of carriage with methicillin-resistant *Staphylococcus aureus*: effectiveness of a national guideline. J Antimicrob Chemother 66:2409–2417. doi:10.1093/jac/dkr243. 21719473

[19] Petersen IS, Christensen JM, Zeuthen AB, Madsen PB. 2019. Danish experience of meticillin-resistant *Staphylococcus aureus* eradication with emphasis on nose-throat colonization and supplementary systemic antibiotic treatment. J Hosp Infect 103:461–464. doi:10.1016/j.jhin.2019.09.005. 31513882

[20] Ammerlaan HSM, Kluytmans JAJW, Berkhout H, Buiting A, de Brauwer EIGB, van den Broek PJ, van Gelderen P, Leenders SACAP, Ott A, Richter C, Spanjaard L, Spijkerman IJB, van Tiel FH, Voorn GP, Wulf MWH, van Zeijl J, Troelstra A, Bonten MJM; MRSA Eradication Study Group. 2011. Eradication of carriage with methicillin-resistant *Staphylococcus aureus*: determinants of treatment failure. J Antimicrob Chemother 66:2418–2424. doi:10.1093/jac/dkr250. 21719471

[21] Bagge K, Benfield T, Westh H, Bartels MD. 2019. Eradicating MRSA carriage: the impact of throat carriage and Panton-Valentine leukocidin genes on success rates. Eur J Clin Microbiol Infect Dis 38:683–688. doi:10.1007/s10096-019-03474-6. 30684163

[22] Lekkerkerk WS, Uljee M, Prkić A, Maas BD, Severin JA, Vos MC. 2015. Follow-up cultures for MRSA after eradication therapy: are three culture-sets enough? J Infect 70:491–498. doi:10.1016/j.jinf.2015.01.006. 25597821

[23] Bernards AT, Bonten MJM, Cohen Stuart J, Diederen BMW, Goessens WHF, Grundmann H, Kluytmans JAJW, Kluytmans-van den Bergh MFQ, Leverstein-van Hall MA, Mouton JW, al Naiemi N, Troelstra A, Vandenbroucke-Grauls CMJE, Vos MC, Voss A. 2012. NVMM Guideline Laboratory detection of highly resistant microorganisms (HRMO), version 2.0. Netherlands Society for Medical Microbiology, Leeuwarden, the Netherlands. https://www.nvmm.nl/media/1051/2012_hrmo_mrsa_esbl.pdf.

